# The Breathe Easier through Weight Loss Lifestyle (BE WELL) Intervention: A randomized controlled trial

**DOI:** 10.1186/1471-2466-10-16

**Published:** 2010-03-24

**Authors:** Jun Ma, Peg Strub, Carlos A Camargo, Lan Xiao, Estela Ayala, Christopher D Gardner, A Sonia Buist, William L Haskell, Phillip W Lavori, Sandra R Wilson

**Affiliations:** 1 Department of Health Services Research, Palo Alto Medical Foundation Research Institute, Palo Alto, CA, USA; 2 Department of Allergy, Asthma, and Immunology, The Permanente Medical Group, San Francisco Medical Center, San Francisco, CA, USA; 3 Department of Emergency Medicine, Massachusetts General Hospital, Harvard Medical School, Boston, MA, USA; 4 Department of Pulmonary Medicine & Critical Care, Stanford University School of Medicine, Stanford, CA, USA; 5 Stanford Prevention Research Center, Stanford University School of Medicine, Stanford, CA, USA; 6 Pulmonary & Critical Care Medicine, Oregon Health & Science University, Portland, OR, USA; 7 Department of Health Research & Policy, Stanford University School of Medicine, Stanford, CA, USA

## Abstract

**Background:**

Obesity and asthma have reached epidemic proportions in the US. Their concurrent rise over the last 30 years suggests that they may be connected. Numerous observational studies support a temporally-correct, dose-response relationship between body mass index (BMI) and incident asthma. Weight loss, either induced by surgery or caloric restriction, has been reported to improve asthma symptoms and lung function. Due to methodological shortcomings of previous studies, however, well-controlled trials are needed to investigate the efficacy of weight loss strategies to improve asthma control in obese individuals.

**Methods/Design:**

BE WELL is a 2-arm parallel randomized clinical trial (RCT) of the efficacy of an evidence-based, comprehensive, behavioral weight loss intervention, focusing on diet, physical activity, and behavioral therapy, as adjunct therapy to usual care in the management of asthma in obese adults. Trial participants (n = 324) are patients aged 18 to 70 years who have suboptimally controlled, persistent asthma, BMI between 30.0 and 44.9 kg/m^2^, and who do not have serious comorbidities (e.g., diabetes, heart disease, stroke). The 12-month weight loss intervention to be studied is based on the principles of the highly successful Diabetes Prevention Program lifestyle intervention. Intervention participants will attend 13 weekly group sessions over a four-month period, followed by two monthly individual sessions, and will then receive individualized counseling primarily by phone, at least bi-monthly, for the remainder of the intervention. Follow-up assessment will occur at six and 12 months. The primary outcome variable is the overall score on the Juniper Asthma Control Questionnaire measured at 12 months. Secondary outcomes include lung function, asthma-specific and general quality of life, asthma medication use, asthma-related and total health care utilization. Potential mediators (e.g., weight loss and change in physical activity level and nutrient intake) and moderators (e.g., socio-demographic characteristics and comorbidities) of the intervention effects also will be examined.

**Discussion:**

This RCT holds considerable potential for illuminating the nature of the obesity-asthma relationship and advancing current guidelines for treating obese adults with asthma, which may lead to reduced morbidity and mortality related to the comorbidity of the two disorders.

**Trial registration:**

NCT00901095

## Background

Obesity has been increasingly linked to asthma. Over the last 30 years there has been a rapid, concurrent increase in the prevalence of both chronic diseases in the U.S. and other westernized countries [1,2]. A growing number of prospective studies support a temporally correct, dose-response relationship between body mass index (BMI) and incident asthma in adults and children of multiple nationalities and racial/ethnic groups [3-5]. Obesity also has been shown to alter prevalent asthma towards a more difficult-to-control phenotype [6-11]. Some evidence suggests that selected manifestations of the obesity-asthma relationship may be stronger in females [12-15]. Animal studies support a causal link between the two disorders [16-18], and several mechanisms have been postulated [5,19-21]. However, elucidating the exact nature of the obesity-asthma association in humans remains an area of active research [20].

The National Asthma Education and Prevention Program Expert Panel Report 3 identified obesity as a significant comorbidity and recommended that treatment of obesity be considered in obese adults with asthma [22]. However, this recommendation was, in the Panel's evaluation, based on limited data. The evidence available to date suggests that weight loss, induced by surgery or caloric restriction, is associated with improved asthma symptoms, lung function, medication usage, and quality of life in obese adults with asthma [23-26]. Much of this research, however, suffers from notable methodological inadequacies, which include lack of randomization, small sample size, and short duration, among others. In addition, neither surgery nor caloric restriction alone is the recommended first-line therapy for weight loss; the latter emphasizes a comprehensive approach to lifestyle change, focusing on diet, physical activity, and behavioral self-management [27]. Further research is needed.

## Aims

The BE WELL (*B*reathe *E*asier through *We*ight *L*oss *L*ifestyle) intervention trial was designed to address the gap in knowledge of whether weight loss improves asthma control. It aims to evaluate the efficacy of an evidence-based, comprehensive weight loss lifestyle intervention as adjunct therapy to usual care in the management of asthma in obese adults. The primary hypothesis is that compared with participants in the usual care (UC) arm, those in the weight loss intervention (WL) arm will achieve greater improvement in asthma control, as measured by a validated composite measure, the Juniper Asthma Control Questionnaire (ACQ) [28], at 12 months follow-up. Secondary aims include comparing the WL and UC arms on changes in ACQ scores at six months and in lung function, asthma-specific quality of life (QoL), asthma medication use, and asthma-related health care utilization (HCU) at six and 12 months. Potential mediators (e.g. weight loss and change in physical activity level and diet composition) and moderators (e.g., socio-demographic characteristics and comorbidities) of the intervention effects will be explored.

## Methods/Design

### Study Design

BE WELL is a two-arm randomized controlled clinical trial (RCT) in which obese patients ages 18-70 years with suboptimally controlled, persistent asthma (n = 324) will be equally randomized to the WL or UC arm. All procedures and materials were approved by the Kaiser Foundation Research Institute's Institutional Review Board.

### Eligibility Criteria

Participants will be recruited from multiple medical centers of Kaiser Permanente in Northern California (KPNC). Patients ages 18-70 years who are obese and have suboptimally controlled, persistent asthma will be eligible to participate. Table 1 enumerates the inclusion and exclusion criteria. Exclusion criteria were designed to ensure each participant's ability and safety to complete the study requirements, minimize error associated with primary outcome, and prevent possible missing data. Individuals of both genders and any racial or ethnic background who speak English, meet the inclusion criteria, and have no exclusion criteria will be eligible. Based on the demographics of obese asthma patients at the selected KPNC sites, the gender and ethnic distributions of the target enrollment population are expected to consist of 67% women, 57% White, 27% Black, 7% Hispanic/Latino, and 9% Asian/Pacific Islander.

**Table 1:** Participant inclusion and exclusion criteria

Inclusion criteria
* Age (as of date of enrollment)
- Lower age limit: 18 years
- Upper age limit: 70 years
* Gender: Both men and women
* Race/ethnicity: All racial and ethnic groups
* Body mass index 30.0-44.9 kg/m^2^
* Suboptimally controlled asthma:
- Documented diagnosis of asthma on the current medical problem list
- Currently prescribed an anti-asthma medication
- Score <20 on the Asthma Control Test [75]
- Demonstrable airway reversibility
* Kaiser member for ≥ 1 year
* Able and willing to enroll and provide written, informed consent, i.e., to: 1) meet the time and data collection requirements of the study; 2) be randomized to one of the two intervention arms; 3) adhere to the recommendations of the study intervention as assigned; 4) participate in follow-up for 12 months; and 5) allow extraction of relevant information from their medical records.
Exclusion criteria
* Inability to speak, read or understand English;
* Intermittent asthma, defined as either seasonal asthma or (daytime asthma symptoms <2×/week and nocturnal symptoms <2×/month and no use of controller medications);
* Diagnosis of COPD (emphysema or chronic bronchitis) suggested by patient report of doctor diagnosis, spirometry, and smoking history;
* Use of weight-loss medications in the past 3 months;
* Initiation or change of antidepressant medication within the past 3 months;
* Regular use (>5 days/month) of the following medications: oral corticosteroids, insulin, oral hypoglycemics, anti-psychotics, and mood stabilizers;
* Currently enrolled in a group or individual intervention program that overtly aimed at weight loss, nutrition and/or physical activity at Kaiser or elsewhere;
* Planning to undergo bariatric surgery during the study period;
* Inability to perform pulmonary function tests by spirometry in a consistent manner;
* Having a medical or physical condition that makes moderate intensity physical activity (like a brisk walk) difficult or unsafe;
* Significant medical co-morbidities, including uncontrolled metabolic disorders (e.g., thyroid, renal, liver), diabetes, heart disease, stroke, and ongoing substance abuse;
* Hospitalization for psychological or emotional problems within the past two years;
* Diagnosis of cancer (other than non-melanoma skin cancer) that is/was active or treated with radiation or chemotherapy within the past 2 years;
* Diagnosis of a terminal illness and/or in hospice care;
* Currently pregnant, lactating, or planning to become pregnant during the study period;
* Already enrolled or planning to enroll in a research study that would limit full participation in the study or confound the interpretation of the study's findings;
* Family/household member of another study participant or of a study staff member;
* No longer a Kaiser patient or planning to transfer care outside of Kaiser during the study period;
* PCP determination that the study is inappropriate or unsafe for the patient;
* Investigator discretion for clinical safety or protocol adherence reasons.

### Recruitment and Screening Process

The target sample size for BE WELL is 324, to be recruited in five sequential cohorts. Recruitment for each cohort will begin by querying KPNC's electronic asthma registry to identify obese adult patients who are likely to have suboptimally controlled, persistent asthma based on them past ER visits, hospitalizations, and/or medication dispensing records. These patients will be reviewed by their primary care provider (PCP) to determine whether their participation in the study is medically appropriate and safe. Patients for whom PCP approval is obtained will be invited by mail to participate in the study and be asked to complete, either online or by phone, a brief eligibility screening questionnaire pertaining primarily to major exclusions that patients can reliably answer on their own (e.g., pregnancy, plans to move, active cancer). Patients who pass the initial screen will be scheduled for a formal in-person eligibility determination visit (BV1) and, if eligible, a second visit (BV2) to complete baseline data collection prior to randomization.

BV1 will occur no more than four months after the initial screen, and no more than two months prior to BV2. Written informed consent will be obtained, followed by completion of additional eligibility questionnaires, including screening for angina, peripheral vascular disease (PVD), and severe depression. Height and weight will be measured and BMI calculated. Patients whose BMI is in the eligibility range between 30.0-44.9 kg/m^2 ^will have spirometry performed before and after administration of a short-acting beta agonist to determine reversibility of airway obstruction. Patients with very poor lung function (pre-bronchodilator forced expiratory volume in one second [FEV1]<40% of predicted or post-bronchodilator FEV1<50% of predicted) or who have possible angina, PVD, or severe depression will be referred to a study physician and their PCP for evaluation and may only continue in the study with explicit physician authorization. Participants who prove fully eligible will be given instructions, a two-week asthma diary form, and a three-day food record form to be completed and returned at BV2.

BV2 will occur at least two weeks after BV1 to allow time for participants to complete the asthma diary and food record. Also, BV2 will occur no more than four weeks prior to the start of the intervention. It will involve review of the patient's two-week asthma diary and three-day food record, completion of the baseline questionnaire, measurement of weight, waist circumference, hip circumference, blood pressure, and pre-bronchodilator spirometry.

### Randomization and Blinding

Following BV2, participants will be randomized in a 1:1 ratio to the WL or UC arm. By applying a balance algorithm that originated in cluster randomized trials [29], BE WELL randomization will assure very close balance at baseline on age, sex, race/ethnicity, BMI and ACQ score stratified by participating KPNC medical centers, and better than chance balance on characteristics correlated with the balancing variables. Within each medical center, participants will be randomized in blocks to take advantage of complete data on baseline covariates across the recruited sample prior to randomization.

For the first block within a medical center, the imbalance scores of all possible allocations with equal numbers of participants between two anonymous groups (1 and 2) will be calculated using equation 1. The correspondence of groups 1 and 2 with either the WL or UC arm will be assigned randomly at the outset and used consistently throughout the trial.(1)

where k is the kth balancing factor (k = 1 to 5) as listed above;  and  are the average of kth factor for groups 1 and 2, respectively; and the weights (w) determine the relative contribution of each balancing factor to the imbalance B [30]. In BE WELL, equal weights will be assumed for all balancing factors. For categorical factors, orthogonal dummy variables, which identify individual factor levels, will be coded before imbalance score calculation. A set of optimal allocations will be determined by the lowest 1,000 calculated B values [30]. One allocation will be randomly selected from the set of optimal allocations for the block of participants about to be randomized.

After randomization of the first block of participants within a medical center, allocations for each subsequent block will be conditional on the chosen allocations of participants in earlier blocks. The imbalance score B for a new block will be computed using equation 2.(2)

where n_1 _is the number of participants in the existing block(s) and n_2 _is the total number of participants with the addition of the new block; *x*_1*ik *_and *x*_2*ik *_represent the kth factor of ith participant allocated in group 1 or 2. The process of sampling from the 1,000 lowest calculated B values will be repeated for all subsequent blocks.

This randomization procedure not only minimizes imbalance for the chosen baseline covariates between treatment groups and correlated characteristics, but also ensures concealment of treatment allocation, with recruitment staff completely unable to influence allocation. A designated research staff member who is not involved in follow-up data collection or data analysis will carry out randomization using a computerized program. Anonymous labels of treatment assignments will be used in all study reports and other materials to ensure blinding of the investigators and staff responsible for follow-up data collection and data analysis. Staff responsible for delivering the WL intervention will be masked to all outcome data until the end of trial. Due to the nature of this intervention, blinding of the participants is not feasible.

### Baseline and Follow-up Measures and Data Collection

Table 2 shows the study measures and data collection schedule. In addition to the screening and baseline data collection activities described previously, follow-up visits for questionnaires, clinical measurements, and adverse event monitoring will be conducted at six and 12 months after randomization.

**Table 2:** List of measures and data collection schedule

	Baseline	Follow-up month	
		
		6	12
**Questionnaires**			
Juniper Asthma Control Questionnaire (ACQ)	X	X	X
Juniper Mini Asthma-specific Quality of Life Questionnaire (mini-AQLQ)	X	X	X
Two-week asthma symptom diary	X	X	X
Baseline Dyspnea Index	X		
Transition Dyspnea Index		X	X
Three-day food record	X	X	X
Eating Habits Confidence Survey	X	X	X
Social Support and Eating Habits Survey	X	X	X
Stanford Seven-day Physical Activity Recall	X	X	X
Exercise Confidence Survey	X	X	X
Total number of steps in the past seven days (per pedometer reading)	X	X	X
Social Support and Exercise Survey	X	X	X
Rose questionnaire for angina and peripheral vascular disease	X	X	X
Center for Epidemiologic Studies Depression Scale (CES-D)	X	X	X
Depression Anxiety Stress Scale	X	X	X
Obesity Related Problems Scale	X	X	X
12-item Short Form Health Survey (SF-12)	X	X	X
Berlin Questionnaire for Sleep Apnea	X	X	X
Gastroesophageal Reflux Disease Symptom Assessment Scale	X	X	X
Adverse events	X	X	X
Care at non-Kaiser health care facilities	X	X	X
Demographics	X		
**Clinical Measures**			
Height	X		
Weight, waist circumference, hip circumference, waist to hip ratio (WHR)	X	X	X
Blood pressure	X	X	X
Spirometry: Forced maneuver - FEV1, FEV6, FVC (absolute and % predicted values), FEV1/FVC, FEV1/FEV6; Slow maneuver - VT, ERV, IC, VCin, VCex	X	X	X
**Data from KPNC Electronic Databases**			
Current medical problems	X		X
Medications currently prescribed	X	X	X
Medications dispensed^2^	X		X
Health care utilization^2^	X		X
Primary care provider characteristics	X		X
**Recruitment and intervention process measures (monitored continuously)**			
**Safety of participants in the intervention arm (monitored continuously)**			

^1^Pre- and post-bronchodilator spirometry will be obtained at baseline visit 1 to determine reversibility of airway obstruction, which is an eligibility criterion. Only pre-bronchodilator spirometry will be performed at baseline visit 2 and at 6- and 12-month assessments and the measurements obtained will be used for analysis.

^2^Data will be extracted for a period of 12 months before and after randomization.

Abbreviations: ERV, expiratory reserve volume; FEV1, forced expiratory volume in one second; FEV6, forced expiratory volume in six seconds; FVC, forced vital capacity; IC, inspiratory capacity; KPNC, Kaiser Permanente in Northern California; VCin and VCex, inspiratory and expiratory vital capacity; VT, tidal volume.

#### Primary and Secondary Outcomes

The primary outcome measure is the Juniper Asthma Control Questionnaire (ACQ). The ACQ provides a reliable and validated measure of the impairment dimension of asthma control [28,31] and includes specific measures of each component of impairment as defined in the current asthma treatment guidelines [22], i.e., daytime and nocturnal asthma symptoms, functional capacity, rescue medication use (excluding use for prevention of exercise-induced bronchospasm), and lung function (FEV1). ACQ is the only composite measure, among the several recommended by the guidelines, that includes lung function in its overall rating and is in widespread use in trials of both asthma pharmacotherapeutic and behavioral interventions [22].

Secondary outcomes include lung function measurements by spirometry (Table 2) and the 15-item Juniper Mini Asthma Quality of Life Questionnaire (mini-AQLQ) [32]. In addition, asthma-related health care utilization (HCU) will be assessed through data extraction from KPNC electronic databases for the period of 12 months pre- and post-randomization. These databases contain information on all outpatient and inpatient HCU at KP facilities, non-KP facilities with which KP contracts for emergency and hospital services, and on reimbursable emergency hospital care at non-contract facilities. Although we will analyze HCU data according to type of encounter, the primary HCU outcome will be the overall rate of outpatient encounters and emergency department and inpatient admissions with asthma as primary diagnosis. Data will also be extracted on current medications prescribed and all asthma, allergic rhinitis, and GERD medications dispensed for the same time periods. In KP, it is estimated that well over 95% of all prescriptions are filled at a KP pharmacy, and patients are very unlikely to have concurrent non-KP sources of asthma care or pharmacy. Therefore, dispensing records provide reliable and unbiased information on medication refill adherence and regimen potency. The former will be assessed by calculating continuous medication acquisition indices (the total days supply of a given type of medication dispensed in a given period of time as a proportion of the total days supply prescribed), for asthma controller and reliever medications separately. The total strength of all controller medications dispensed in a given time period will be expressed in beclomethasone-80 canister equivalents, and the total strength of all reliever medications dispensed in a given time period will be expressed in albuterol canister equivalents, as we have done in our previous research [33]. For combination medications (e.g., fluticasone-salmeterol), each active ingredient component of the medication will be counted separately and all will contribute to the adherence and potency calculations.

Additional secondary outcomes include measurements of waist circumference, hip circumference, and resting blood pressure according to standardized protocols as recommended by the PhenX project https://www.phenx.org). Dyspnea with activities of daily living will be assessed using a baseline dyspnea index to rate severity of dyspnea at a single state and a transition dyspnea index that denotes changes from that baseline [34]. The 12-item Short Form Health Survey (SF-12) [35], a widely used measure of generic health-related QoL, will be administered to evaluate changes in non-disease specific physical and mental health status. The Obesity-Related Problem Scale is a reliable eight-item scale that measures the impact of obesity on psychosocial functioning and is responsive to weight loss intervention [36]. Mental health status will be assessed using the 20-item Center for Epidemiological Studies Depression scale (CES-D) [37] and the Depression Anxiety Stress Scale [38]. In addition to asthma-related HCU, utilization for other obesity-related co-morbidities, non-asthma pulmonary diagnoses, and all other HCU will be assessed.

#### Potential Mediators

The intervention to be studied involves changes in diet and physical activity aimed at producing weight loss. Undoubtedly, there will be variation in changes in weight loss, physical activity level, and diet. Also, some evidence suggests that physical activity and certain nutrients may have independent effects on asthma outcomes [39-44]. Investigating the relative contribution of changes in BMI, physical activity, and diet will provide evidence of whether the underlying mechanism for improvement in asthma control is related to weight loss *per se *or not. Caloric and nutrient intake will be determined from food records collected on two non-consecutive weekdays and one weekend day within a seven-day period at each assessment point. Multiple-day food records are considered a gold standard for collection of individual dietary data [45]. Food composition will be analyzed using the Nutrition Data System. Physical activity will be measured using the Stanford 7-Day Physical Activity Recall, which is a reliable measure that is sensitive to change in physical activity [46-48].

#### Potential Moderators

To elucidate differences in intervention effects among subgroups of the study sample, we will collect data on important socio-demographic characteristics: age, sex, race/ethnicity, education, marital status, household income, medical insurance, smoking status, and age at onset of asthma symptoms. In addition, the Berlin Questionnaire for sleep apnea[49] and the Gastroesophageal Reflux Disease (GERD) Symptom Assessment Scale[50] will be used to screen for possible sleep disorders and GERD, respectively, as these are common comorbidities in obese individuals with or without asthma. Their presence may modify the intervention effect.

#### Process Measures

Data to assess the likelihood of potential subsequent adoption of the weight loss intervention by the health care system, such as the representativeness of physician approval of their patients' participation, patient willingness to enroll in the study, and reasons for declining to participate will be documented. Intervention process measures will include dates and number of classes attended, number and duration of in-clinic visits and phone calls, patient self-monitoring records, and data electronically stored in pedometers provided to participants by the study.

### Interventions

#### Theoretical Basis

The theoretical underpinning of the BE WELL weight loss intervention is derived from Social Cognitive Theory [51] and the Transtheoretical Model of Behavior Change [52]. The former emphasizes the reciprocal determinism between individual, environment, and behavior, whereas the latter recognizes that behavior change is a dynamic process that moves, at variable speed, through stages of readiness to change. Behavior change is more likely with increased behavior capability, and behavior capability is strengthened through goal setting, skill building and self-monitoring [51]. Also important are confidence in one's ability to perform a given behavior (self-efficacy) and expectation of favorable outcomes of the behavior (outcome expectations). Behavioral strategies may vary by stage of change (i.e., experiential processes during initial phases of behavioral adoption and behavioral processes occurring during action and maintenance phases) [52]. In addition, the BE WELL intervention draws upon key research evidence for the importance of self-management in chronic disease management [53-55]. Successful self-management intervention is driven by patient-defined problems and fosters the mastery of skills in problem solving, action planning, decision making, and support building through an iterative process [55,56].

Furthermore, the BE WELL intervention is based on the premise that long-term changes in diet and exercise and sustained motivation to maintain behavior changes are most likely to occur when the strategies used to motivate and support behavior change are flexible, sensitive to cultural and individual differences, and broadly acceptable to those in the target population. We make use of a combination of a structured protocol (in which all participants receive certain common core information) and individually tailored strategies to help each participant achieve and maintain specific physical activity and dietary changes directed toward the overall intervention goals of losing weight and increasing physical activity.

The BE WELL intervention clearly differs from the delivery of lifestyle modifications in the usual primary care setting. Current practice routinely involves physician advice, occasionally with referral to a dietitian who takes a diet history and who then provides standard educational materials and advice during a small number of individual or group sessions, typically with little or no follow-up. This standard clinical approach lacks the theoretical basis and behavioral self-management focus described above.

#### Evidence-based Intervention Goals

A goal-based approach is used in the BE WELL intervention in which participants will be given the same weight loss and physical activity goals. The weight goal is to achieve and maintain a weight loss of 7% of baseline body weight in a gradual stepwise fashion. A 7% weight loss has proven to be feasible and safe and is associated with clinically significant reductions in the risk of diabetes and cardiovascular disease [57-60]. Participants who wish to lose more than 7% of their starting weight may be encouraged to do so only insofar as the participant maintains a normal BMI at or above 21 and the rate of weight loss does not exceed three pounds per week. The physical activity goal is to achieve and maintain a minimum of 150 minutes per week of moderate intensity physical activity (such as brisk walking). This goal is consistent with the new Physical Activity Guidelines for Americans [61], and is deemed safe and attainable for most adults, including those with chronic conditions such as asthma. Participants, especially those who are habitually sedentary, will be advised to gradually and steadily increase daily walking with a goal of achieving 150 minutes of brisk walking per week by the end of the Intensive intervention stage in month 4. Regular physical activity recommendations for activities of moderate intensity other than brisk walking, as well as strength and flexibility physical activities, will be tailored to the participant's situation [62,63]. After attaining the minimum goal of 150 minutes per week, participants who wish to be more active will be encouraged to do so, as tolerated. If participants reach the 150-minute goal but are not achieving the weight goal, they will be encouraged to further gradually increase their physical activity to 60 minutes/day of moderate physical activity [62,64,65] as well as to consider further calorie restriction.

To help them achieve and maintain the weight goal, participants also will be given a goal for daily calories. A moderate reduction of caloric intake by 500-1000 kcal/day has been shown to produce clinically significant weight loss over time and is a safe and achievable goal for most people [27]. Of note, the calorie goals will be given as a means to achieve and maintain the weight goal, rather than as a goal in and of itself. Therefore, if a participant consumes more than the assigned calorie goal, but is achieving the weight goal, there is no need to focus on further reductions. Participants will be encouraged to gradually achieve the calorie goals through portion control, choices of low-energy and nutrient-dense meals and snacks, healthy food preparation techniques, and careful selection of restaurants, including fast food, and of the items offered. Participants also will be advised to: (1) lower saturated fat intake to <10% of caloric intake, (2) lower cholesterol intake to <300 mg/day, (3) consume a high plant-based diet that includes a variety of vegetables, beans, whole grains, low-fat dairy products, and whole fruits, and (4) reduce intake of high glycemic index carbohydrates.

#### Intervention Format, Structure, and Content

Participants in both the UC and WL arms will continue to receive the standard of medical care from their PCPs and any specialists, per KPNC treatment protocols. In addition, they will receive an Omron HJ-7201ITC pedometer, which records steps taken, and an HBF-400 scale for home weight measurement. They will also receive KPNC standard asthma self-management and weight management educational handouts and a list of elective weight management services offered at the participating medical centers.

In addition to usual care, participants in the WL arm will progress through three successive intervention stages: Intensive, Transitional and Extended stages. Table 3 shows the design elements of the BE WELL intervention during the three stages.

**Table 3:** Design elements of the BE WELL weight loss intervention

Stage	Intensive (Months 1-4)	Transitional (Months 5-6)	Extended (Months 7-12)
**Goal of contact**	Gradual weight loss associated with small, progressive changes in diet and physical activity		Weight maintenance or continued gradual weight loss
**Contact schedule**	Weekly (13 contacts)	Monthly (2 contacts)	Bi-monthly/monthly (typically 3-6 contacts)
**Contact mode**	Group, in person (Family member welcome)	Individual, in person (Family member welcome)	Individual, by phone (in person as needed)
**Contact duration**	90-120 minutes	30-60 minutes	Variable
**Contact structure**	* Private weigh-in * Review and sharing of progress, or lack thereof, in relation to action plan/goals from last session * Didactic presentation of a new topic * 30-minute supervised physical activity session * Development of individual action plan/goals for the next week	* Private weigh-in * Review of self-monitoring records * Identification of personal barriers to weight loss and activity * Problem solving and relapse prevention * Development/revision of a customized care plan	* Review of self-monitoring records * Detection of signs of non-adherence and relapse * Problem solving to encourage adherence and prevent relapse * Revision of the customized care plan * If indicated, re-initiation of intensive weight loss efforts through individual or small group visits

##### Intensive Stage

The Intensive stage of the BE WELL intervention is adapted from the Group Lifestyle Balance (GLB) program™ [66]. The GLB program is based on the highly successful Diabetes Prevention Program [57] and includes 12 weekly small group sessions. Each session follows a similar structure and includes five curriculum components: 1) measurement and recording of weight, 2) review of self-monitoring records and progress, 3) identification of personal barriers to weight loss and activity and potential solutions, 4) presentation of a new content area, and 5) goal setting and action plans for next week. In BE WELL, the GLB Manual of Operations will be closely followed, except for the addition of a food tasting activity as part of the check-in process and a 30-minute segment of physical activity demonstrations and practices at the end of each session. The food tasting activities are aimed at increasing participant experience with healthy food choices, encouraging them to try new foods that they might not normally try on their own, and stimulating a social, fun and engaging environment. The physical activity demonstrations and practices are aimed at increasing participant awareness of physical activity intensity, experiencing various moderate-intensity activities that are safe and that they can easily incorporate into their daily lives, and increasing participant confidence. As part of these supervised exercises, participants will learn how to recognize exercise-induced bronchospasm based on symptoms and peak flow meter readings and the appropriate prophylaxis and treatment. In BE WELL, participants will be given a pedometer and start using it to record their daily steps in week 1, as opposed to starting this in week 10 as in the original GLB program. Furthermore, BE WELL participants will attend an additional group session focusing on the latest evidence that came out after the GLB program on high-carbohydrate and high-fat/high-protein dietary approaches to weight loss to complement the program's emphasis on low-fat diets. The 13 group sessions of 90-120 minutes each will be delivered by a nutrition health educator and a fitness instructor over a 4-month period.

##### Transitional Stage

In the Transitional stage, participants will attend one individual counseling session in month 5 and another one in month 6. The goals of these visits are to 1) finalize a customized, comprehensive action plan for weight loss and maintenance viable in the participant's environment, and 2) facilitate participant independence in carrying out and maintaining that behavior change as the influence of the interventionists diminishes. During the sessions, interventionists will review and discuss the participant's self-monitoring records of weight, dietary intake and physical activity, provide feedback on progress (or lack thereof), discuss barriers to behavior change, and will advise on problem solving and relapse prevention strategies.

##### Extended Stage

The goal of the remaining 6 month will be to facilitate the continued evolution of behavior changes achieved to date and to promote long-term weight stability. Participants will continue to monitor their weight, dietary intake, and physical activity based on their care plan. Interventionists will monitor the progress of each individual and provide individualized feedback and structured guidance by phone at least bi-monthly. Participants may initiate contact with the interventionists at any point. At the discretion of the interventionists, participants may be seen in person to address outstanding issues related to poor understanding of and/or adherence to the intervention protocol. Participants with weight gains of 3 to 5 lbs (1.4 to 2.2 kg) will be telephoned on a biweekly basis until they return to a stable, lower weight. A weight gain of 5 lbs (2.3 kg) or more will automatically trigger a suggestion of an in-clinic visit at which the participant will be encouraged to restart or intensify active weight-loss efforts and a new action plan will be created to guide the efforts.

### Participant Safety

Participants will be carefully screened, and individuals for whom the intervention is deemed medically inappropriate or unsafe will be excluded. PCP approval will be required before potentially eligible patients are contacted by the study. During screening, women who are pregnant, lactating, or planning to become pregnant during the study period will be excluded. If a participant becomes pregnant during the study, she will be excluded from further participation in all study activities, and her PCP will be notified. Participants who are diagnosed with any other exclusionary condition (e.g., coronary heart disease, stroke, diabetes, cancer) following randomization may continue with the intervention (if applicable) and follow-up assessments only after explicit approval of a study physician.

Surveillance for adverse events and relevant clinical events will occur by questionnaire at regularly scheduled assessment points and by systematic query of all participants' electronic health records at three month intervals throughout the follow-up. Similar information reported by participants at other times (e.g., during intervention encounters) will be duly noted and followed up to assure participant safety. A study physician will review all adverse event reports to determine whether they are serious, study related, or expected, and the events will be reported to the IRB, DSMB, and the sponsor according to the reporting timelines appropriate to the type of the event.

Alert levels (e.g., for lung function and blood pressure) and alert conditions (e.g., asthma exacerbations, cardiovascular events, musculoskeletal injuries) and referral protocols have been established to ensure that participants are offered appropriate evaluation and therapy when clinically indicated. In all cases, participants may be immediately referred for medical evaluation as needed or recommended by the study clinician.

### Retention

Adherence and retention in BE WELL is expected to be fostered by: 1) selection/retention of qualified staff, their training, and systematic quality control; 2) the relationship between staff and participants, 3) willingness to accommodate participant schedules and needs, and 4) resourcefulness and persistence of the staff. For purposes of follow-up assessment, a participant tracking database will be used to assure that participants are contacted on a timely basis. Subjects' contact information will be kept current and at least two alternative contacts will be collected. Subjects will be given a nominal payment for each data collection visit. Assessment staff, who will be blinded to treatment assignment, will contact subjects who miss a visit to reschedule the visit and to re-engage them in subsequent follow-ups. Subjects will be educated about the importance of follow-up assessment even if they are not adhering to the treatment. Priorities will be given to the primary and main secondary outcomes in cases where not all follow-up measurements may be obtained from a subject.

Ongoing monitoring of indices of participation in intervention activities helps interventionists identify participants who are having problems adhering to the intervention protocol and thus initiate recovery efforts. Such efforts can range from brief telephone discussion to an individual counseling session with the interventionist in which the participant who makes a decision to discontinue intervention can review concerns regarding BE WELL, reasons for staying in the study, and decisions about remaining a participant. Unless the participant declines, the interventionist will continue to contact the participant on a progressively less frequent schedule, starting monthly and decreasing to semi-annually to remind her/him of the opportunity to re-enter BE WELL.

### Sample size consideration and statistical analysis

#### Sample Size

In the only RCT conducted to date of lifestyle-induced (by caloric restriction and behavior change) weight loss for the management of asthma, effect size (Cohen's d) estimates ranged between 0.4 and 0.6 for various asthma symptom and lung function outcomes over a 12-month follow-up period [26]. A sample size of 324 is sufficient to detect an effect size of 0.4 between the WL and UC arms for 90% power at 5% alpha 2-sided, assuming up to a 20% loss to 12-month follow-up (or an end-of-the-study sample size of 259). The power estimate was calculated based on an independent t-test using simplified assumptions. Note that the t-test calculation is conservative because it does not account for variance reduction due to adjustment for repeated measures, nor for the better than chance comparability of the experimental groups.

#### Statistical analysis

The primary hypothesis compares Juniper ACQ scores between the WL and UC arms at 12 months using an intent-to-treat analysis. Linear mixed modeling, using SAS PROC MIXED [67] will test time and group interactions to assess whether within-subject change in Juniper ACQ scores differs by condition (equation 3).(3)

Due to randomization, the distributions of baseline values on the dependent variable (Y_0_) and baseline covariates (X_i _where i = 1 to k) should be similar between treatment arms and thus not bias the analysis. Nonetheless, to the extent they are associated with the outcome, their inclusion in the model will account for otherwise unexplained variation and hence increase the efficiency of the analysis. In addition, their inclusion will guard against imbalance in the distributions of these covariates between treatment arms, which can still occur despite randomization. a, g, and δ represent the random effects caused by clustering of patients with physicians and, in turn, physicians within clinics, and by blocking of patients within medical centers. ε takes into account the correlation of repeated measures within subjects.

The same analysis strategy is appropriate for evaluating secondary aims where the hypotheses are identical but with different outcome measures (e.g., lung function, asthma-specific QoL, asthma-related HCU). The longitudinal effects of the intervention will be assessed using the same model, except that the focus is comparisons within the intervention arm at different time points. Analyses will also be conducted to examine whether there is a dose-response relationship between intervention adherence and outcome, where dose is defined in terms of number of group sessions attended, number of self-monitoring logs kept, average daily intakes of calories and fat, and average number of minutes of physical activity per week.

The primary analyses will be intention-to-treat. Secondary per-protocol or as-treated analyses will be performed to help elucidate the primary findings. For subjects with missing data at follow-up, we will conduct and report on sensitivity analyses in which several previously recommended approaches for handling missing data in longitudinal trials will be used, e.g., multiple imputation and the pattern-mixture model [68].

We will use the MacArthur approach, which modified the Baron & Kenny criteria, for defining moderators and mediators in clinical research studies [69]. Let T be the treatment, M be the potential moderator or mediator, and O be the outcome. Association between T, M, and O is examined using the following models:(4)

M can be considered a mediator of the effect of T on O if T precedes M, γ1 ≠ 0, and either β2 ≠ 0 or β3 ≠ 0. M is a moderator of the effect of T on O if M precedes T, γ1 = 0, and β3 ≠ 0.

### Data Management

All study data will be entered into computerized data files utilizing: 1) Microsoft Access for participant tracking and intervention data entry, 2) Vovici's survey software (Dulles, VA) for self-administered questionnaires and entry of clinical measurements, 3) the Nutrition Data System for nutrient analysis based on three-day food records, and 4) a custom-designed web application for seven-day physical activity recall data entry. Research assistants (blinded to participant group assignments) will review participant responses on questionnaires with participants present, and skipped or incorrectly addressed items will be brought to the participant to correct. All of the data entry systems employ automatic, real-time range, logic, and missing value checks. Data sets will be cleaned, verified and archived, and then read into SAS (Enterprise Guide 4.1; SAS Institute, Cary, NC) data sets, which also will be archived. One official copy of all the study data and a master data dictionary will be maintained and updated regularly by the study data analyst. All analytic and tracking database files will be stored in a secure network drive with daily backups. One copy of the backups will be saved on-site and one off-site. Separate archival databases will be permanently maintained. The data files can be shared by authorized study personnel both on-site and in remote locations via a secure virtual private network. Data security will be ensured through password protection and data encryption.

Protected health information will be used and disclosed in accordance with the HIPAA regulations.

### Treatment Fidelity

Intervention quality assurance procedures ensure that project activities are standardized across the interventionists and across the cohorts and that intervention process data are collected accurately. To achieve this, standardized protocols, procedures, and educational materials are prepared, and staff are systematically trained in their use. Provider and auditor checklists are created to ensure that the intervention protocols are followed and that treatment objectives for each group session are met. Interventionists will complete a Provider Checklist after each session. All group and individual sessions will be audiotaped and a random sample of 10% selected for rating by a qualified individual using the Auditor Checklist. When a session is reviewed with less than 85% of treatment-specific objectives met, the auditor will inform the PI who will remediate interventionist training as needed. This process will continue through all treatment waves so that interventionist drift can be swiftly and consistently corrected.

## Discussion

On a population basis, the high prevalence of obesity among adults with asthma suggests an important opportunity to improve the lives of many such individuals. While the clinical treatment of asthma should continue to emphasize implementation of proven pharmacological and patient self-management strategies [22], the available evidence is already sufficient to warrant the investigation of including evidence-based weight loss strategies as an adjunct therapy for asthma patients who are also obese. Intensive weight loss interventions that employ a combination of recommended dietary, physical activity, and behavioral strategies have consistently resulted in weight losses in the range of 5-7%, which are associated with significantly reduced risk of well-recognized co-morbidities of obesity, such as diabetes and hypertension [57-60]. The efficacy of such a comprehensive, evidence-based, lifestyle approach to weight loss in improving asthma outcomes has not been investigated for obese individuals with asthma.

In BE WELL, the primary objective is to investigate whether weight loss, resulting from dietary modification, physical activity and behavior change, has significant clinical benefits for improving asthma control in obese adults. To our knowledge, BE WELL is the first study with adequate design and power for investigating the longitudinal effects of a behavioral weight loss intervention on asthma control. The only RCT for a similar purpose published to date was conducted by Stenius-Aarniala et al. [26] with the sample of 38 adult obese asthmatics. The results showed a net mean weight loss of 9.1%, which was associated with significant improvements in self-reported asthma symptoms and objectively assessed lung function (by spirometry) over 12 months. Other, non-randomized studies reported that weight loss induced by surgery [70-72] or dietary restriction [23,24,26,73] was associated with improved asthma symptoms, lung function, medication use, quality of life, and health care utilization.

The targeted clinical population in BE WELL is important from the standpoint of its risk for poor asthma outcomes, as well as metabolic and cardiovascular disease. For such individuals, weight loss through intensive lifestyle intervention, rather than surgical intervention or dietary restriction alone, is recommended as first-line therapy. Weight loss is recommended for obese patients with asthma by The National Asthma Education and Prevention Program Expert Panel Report 3 [22], but a lack of strong evidence of the efficacy of weight loss for improving asthma control was recognized by the Expert Panel. BE WELL will provide objective evidence on whether a state-of-the-art weight loss lifestyle intervention achieves a statistically and clinically meaningful improvement over a year's follow-up in asthma control and other manifestations that EPR3 considers indicative of current impairment (symptoms, lung function, rescue medication use, asthma-specific quality of life) and future risk (asthma exacerbations and medication side effects).

Achieving the goals of this study holds considerable potential for advancing the understanding of the causality of the obesity-asthma association and identifying effective behavioral approaches for the management of asthma in adults with comorbid obesity. This study will also serve as a foundation for future studies aimed at understanding the mechanisms by which weight loss is expected to improve asthma control, which may also illuminate the mechanism(s) linking obesity and the development of asthma. Future research is also needed to investigate the effect of weight loss on asthma control among children - a significantly under-researched area even compared to the limited research in adults [74]. Findings from this RCT, if as projected, will strengthen current guidelines for treating obese adults with asthma, which may lead to reduced morbidity and mortality related to both chronic diseases.

## Competing interests

Dr. Camargo has received financial support from a variety of groups for participation in conferences, consulting, and medical research. During 2005 to 2010, industry sponsors with an interest in asthma were AstraZeneca, Critical Therapeutics, Dey, Genentech, GSK, Merck, Novartis, Respironics, and Schering-Plough. Dr. Buist is a member of advisory boards for AstraZeneca, Merck, GSK, Sepracor, Pfizer and Novartis.

All other authors declare that they have no financial, research, organizational, or other interests to disclose that are relevant to the execution of this research or this publication.

## Authors' contributions

JM conceived of the study, has the overall responsibility for its design and conduct, and drafted the manuscript. LX and EA contributed to drafting the manuscript and LX also contributed to the design of the statistical analysis. PS, CAC, CDG, ASB, WLH, PWL, SRW participated in the design of the study and revised the manuscript critically for important intellectual content. All authors read and approved the final manuscript.

## Pre-publication history

The pre-publication history for this paper can be accessed here:

http://www.biomedcentral.com/1471-2466/10/16/prepub
